# Editorial: Protein kinase inhibitors in neurodegeneration and cancer targeted therapies

**DOI:** 10.3389/fcell.2024.1413293

**Published:** 2024-04-18

**Authors:** Saleha Anwar, Azaj Ahmed, Vasiliki Sarli, Imtaiyaz Hassan

**Affiliations:** ^1^ Department of Toxicology, School of Chemical and Life Sciences, Jamia Hamdard, New Delhi, India; ^2^ Department of Internal Medicine, University of Iowa, Iowa City, IA, United States; ^3^ Laboratory of Organic Chemistry, Department of Chemistry, Aristotle University of Thessaloniki, University Campus, Thessaloniki, Greece; ^4^ Centre for Interdisciplinary Research in Basic Sciences, Jamia Millia Islamia, New Delhi, India

**Keywords:** protein kinase, cancer therapies, neurodegenenerative diseases, mutation, cancer

Protein kinases play roles in diverse cellular functions, with approximately 2% of the human genome dedicated to encoding them. The overexpression or mutation of these kinases is associated with cancer development processes (Kannaiyan and Mahadevan, 2018). Targeting kinases with oncogenic transformational potential and involvement in metastasis has significantly altered the clinical approach to cancer management. Numerous kinases, numbering in the hundreds, participate in overlapping and complex processes such as cancer initiation, proliferation and malignancy. Kinases present a challenge in cancer and various disorders such as neurodegenerative disorders (Dell’Aversana et al., 2024; Sengupta et al., 2024; Song et al., 2024). In this Research Topic, we explored the involvement of kinases in the initiation and advancement of different cancers and neurodegenerative disorders. Specifically, we examine the latest significant contributors linked to the onset and progression of these conditions, offering insights and suggesting avenues for future research aimed at enhancing our comprehension of kinase dysregulation in both cancer and neurodegenerative diseases.

## Kinases associated with cancer and neurodegeneration

Protein kinases are the modulators of various signaling cascades and govern major functions of cellular homeostasis. Kinase overexpression is associated with various human diseases (Borgo et al., 2021; Lee and Paull, 2021), including cancers and neurodegenerative disorders (Yadav et al., 2021). Recent findings have unravelled kinases as therapeutic markers and urged the development of potent and selective inhibitors against kinases.

## p90 ribosomal S6 kinase

p90 ribosomal S6 kinase (RSK) is a Ser/Thr protein kinase that acts as a downstream mediator of extracellular signal-regulated kinase 1/2 (ERK1/2), becoming activated following signaling from either Tyr-kinase receptors or GPCRs. RSK modulates various processes associated with cellular homeostasis. RSK has been linked to various cancers, particularly investigated within transformation and metastasis. However, the progress in developing dedicated RSK inhibitors for cancer treatment has been slower than other MAPK signaling pathway components. Wright and Lannigan have highlighted the role of RSK in cancer and assessment of RSK isoform expression levels has received thorough scrutiny across different cancer types, and a summary of these protein expression levels is provided. Progress in crafting dedicated RSK inhibitors for cancer treatment has fallen behind. A potential hurdle in advancing RSK inhibitor candidates to clinical application lies in creating inhibitors specific to each isoform, a daunting task given the striking similarity among their N-terminal kinase domains (NTKDs). While C-terminal kinase domain (CTKD) inhibitors have restricted therapeutic applicability due to their inability to target NTKD activity directly, they could find utility in the design of proteolysis-targeting chimeras.

## Receptor tyrosine kinase-like Orphan Receptor 1

Breast cancer (BC) is the most prevalent form of cancer among women, with up to 15% of cases categorized as triple-negative BC (TNBC). Receptor tyrosine kinase-like Orphan Receptor 1 (ROR1) has become a major therapeutic target against TNBC. Gupta et al. screened seventy thousand chemicals against ROR1 using AutoDock Vina and Glide. Ten representative compounds were obtained through consensus voting, structural alert deletion, and clustering. Compound (CID1261330) showed the best docking score with ROR1 and interacted in the active pockets with various interactive forces.

Further stability of the protein-ligand complex was assessed with molecular dynamic simulations. The compound showed anti-proliferative effects on various breast cancer cell lines, with IC50 values ranging between 2 μM and 10 μM. The results of this study showed the selected compound as a potent inhibitor of ROR1, demonstrating its broader applicability as a proof-of-concept.

## Interleukin-1 receptor-associated kinase 4

The Interleukin-1 receptor-associated kinase 4 (IRAK4) kinase occupies a pivotal position in cellular signaling, crucial for cancer cell survival through various pathways such as activating and translocating NF-κB for inflammatory responses and regulating interferon-α/β receptor in innate immune signaling. Consequently, the inhibition of IRAK-4 has garnered significant interest in recent years, spanning indications from oncology to autoimmune disorders and neurodegeneration, among others. Parrondo et al. provided an extensive review of the existing pre-clinical and clinical data on IRAK-4 inhibitor, emavusertib in managing relapsed/refractory (R/R) B-cell lymphomas and myeloid malignancies.

## Activin receptor-like kinase 3

Activin receptor-like kinase 3 (ALK3), a transmembrane receptor, is associated with BMP signalling. Recent investigations have highlighted the role of ALK3 in mineralized tissues. Deletion or mutation of the kinase has been linked to skeletal anomalies and impediments in tooth development, eruption, and movement. Ruan et al. delineate the function of ALK3 in regulating mineralized tissues and highlight the role of ALK-signaling in bone and teeth physiology. Moreover, this study provides a basis for suggested basic research and possible future therapeutic approaches for renewing and regenerating mineralized tissues.

## Putative markers in Glioblastoma multiforme microenvironment

Remarkably, Kumari and Kumar underscored the importance of genes, such as BMP1, LOX, PLOD1, SERPINE1, etc., in developing GBM. Additionally, their investigation unveiled a positive relationship between E2 conjugating enzymes (Ube2E1, 2H, 2J2, 2C, 2J2, and 2S), E3 ligases (VHL and GNB2L1), and the substrate HIF1α. Moreover, new acetylation sites introduced by HAT1 were identified for Ube2S (K211) and Ube2H (K8, K52). Analysis of the structure and function of Ube2S (8) and Ube2H (1) illuminated their connections with protein kinases, which hold significant implications in developing GBM.
